# Identifying Psychological Symptoms Based on Facial Movements

**DOI:** 10.3389/fpsyt.2020.607890

**Published:** 2020-12-15

**Authors:** Xiaoyang Wang, Yilin Wang, Mingjie Zhou, Baobin Li, Xiaoqian Liu, Tingshao Zhu

**Affiliations:** ^1^Institute of Psychology, Chinese Academy of Sciences, Beijing, China; ^2^Department of Psychology, University of Chinese Academy of Sciences, Beijing, China; ^3^School of Computer Science and Technology, University of Chinese Academy of Sciences, Beijing, China

**Keywords:** mental health, psychological symptoms, SCL-90, facial movements, machine learning, multitrait-multimethod matrix

## Abstract

**Background:** Many methods have been proposed to automatically identify the presence of mental illness, but these have mostly focused on one specific mental illness. In some non-professional scenarios, it would be more helpful to understand an individual's mental health status from all perspectives.

**Methods:** We recruited 100 participants. Their multi-dimensional psychological symptoms of mental health were evaluated using the Symptom Checklist 90 (SCL-90) and their facial movements under neutral stimulation were recorded using Microsoft Kinect. We extracted the time-series characteristics of the key points as the input, and the subscale scores of the SCL-90 as the output to build facial prediction models. Finally, the convergent validity, discriminant validity, criterion validity, and the split-half reliability were respectively assessed using a multitrait-multimethod matrix and correlation coefficients.

**Results:** The correlation coefficients between the predicted values and actual scores were 0.26 and 0.42 (*P* < 0.01), which indicated good criterion validity. All models except depression had high convergent validity but low discriminant validity. Results also indicated good levels of split-half reliability for each model [from 0.516 (hostility) to 0.817 (interpersonal sensitivity)] (*P* < 0.001).

**Conclusion:** The validity and reliability of facial prediction models were confirmed for the measurement of mental health based on the SCL-90. Our research demonstrated that fine-grained aspects of mental health can be identified from the face, and provided a feasible evaluation method for multi-dimensional prediction models.

## Introduction

Mental illnesses have a significant impact on an individual's physical health (1), achievements (2, 3), and life satisfaction (4). In addition to scales, behavioral recognition methods have been developed to judge the existence (5) or degree (6, 7) of specific mental illnesses. However, identifying an individual's mental health status from a range of perspectives may be more helpful in non-professional scenarios such as self-monitoring or large-scale monitoring.

Many studies have found that the physiological and behavioral indicators of individuals with mental illnesses differ, including brain activity (8, 9), galvanic skin response (10), eye contact (11, 12), voice (13, 14), and facial movements (15). Moreover, people with different mental health disorders behave differently (16, 17). For example, patients with schizophrenia can be distinguished from those with depression by analyzing their non-verbal behavior during medical consultation (16). More granularly, neural activity in response to different emotional faces can help distinguish bipolar depression from unipolar depression. Such differences make it possible for machine learning models to diagnose the multi-dimensional psychological symptoms of mental illnesses. Meanwhile, the Symptom Checklist 90 (SCL-90) (18) provides a simple way for researchers to obtain a series of quantitative indicators to comprehensively describe an individual's mental health.

Of all the non-verbal cues related to mental health, facial expressions are relatively stable (19) and easy to obtain. Consequently, we used facial prediction models based on SCL-90 to assess the psychological symptoms of mental illnesses. Given that this is a multi-dimensional research, one model should predict the same symptomatic dimension as assessed by the corresponding subscale, meaning that the depression model and the depression subscale should measure the same thing. Existing model evaluation methods, such as accuracy or mean square error, cannot evaluate such convergent validity. Therefore, we applied the assessment method of scales to machine learning models. The development and application of scales are typically accompanied by tests of reliability and validity. Researchers use the correlation between the scores of a certain scale with those of other scales to evaluate the criterion validity, convergent validity, and discriminant validity, and use the correlation between the scores of the two half items in the scale to evaluate the reliability (20, 21). Similarly, we used the correlation between the predicted scores from models and actual scores from scales to calculate validity, and used the correlation between predicted scores from models based on the two halves of the facial data to test reliability.

In summary, we obtained facial movements and SCL-90 scores, built facial prediction models to identify psychological symptoms, and calculated reliability and validity by way of evaluation. The results showed that our method has fair reliability and validity, and revealed the possibility for machine learning models to recognize more detailed aspects of mental health status, not just at the disease level.

## Materials and Methods

### Participants

We recruited participants at a large event in Wuhan in July 2019, most of whom were coach drivers. The exclusion criteria for this study included: (1) participants whose scale scores were all minimum or maximum; (2) participants whose facial data recorded by Kinect were <700 frames. After balancing gender and normalizing the SCL-90 score distribution, 100 participants were included in the final analysis, including 60 males and 40 females.

### Instruments

*Demographic information*. Basic demographic information such as the gender, age, number of children, education level, and marital status of each participant was obtained.

*Symptom check list*. The SCL-90 (18) is a 90-item self-report scale with responses made on a 5-point Likert scale. It was first used in China in 1984 (22). The SCL-90 assesses mental health status over the past seven days, using 10 subscales reflecting 10 physical and psychological symptoms. Since the SCL-90 assesses a wide range of psychiatric features and can measure multiple physical and psychological symptoms, it has been widely used in the mental health assessment of various groups (23). Due to the limited data collection time available, we chose the six symptomatic dimensions of the SCL-90 which contribute most to people's mental status (24–29), and are also known to affect the non-verbal expression of individuals (30–34). Those dimensions were: interpersonal sensitivity, depression, anxiety, hostility, phobic anxiety, and psychoticism. A brief descriptive summary of each of the six symptoms is provided in Table 1 (35). It is generally believed that when the factor scores of the SCL-90 are >=2, the individual suffers from negative mental health symptoms (factor score = subscale score/number of items). As a result, the threshold of the total score of six symptomatic dimensions was equal to 110 points in this study.

**Table 1:** Details of the six dimensions of SCL-90 used in this study.

**Dimension**	**Number of items**	**Score interval**	**Dimension description**
Interpersonal sensitivity	9	9–45	This scale assesses feelings of insufficient personal abilities and negative expectations of interpersonal interactions.
Depression	13	13–65	This scale assesses a depressed mood, loss of motivation, and suicidal tendency.
Anxiety	10	10–50	This scale reflects symptoms and behaviors related to anxiety.
Hostility	6	6–30	This dimension includes thoughts or behaviors of an emotional state of anger.
Phobic anxiety	7	7–35	This scale refers to persistent anxiety about a specific person, place, object, or sitting posture.
Psychoticism	10	10–50	This dimension provides a graduate rank from mild interpersonal alienation to serious mental illness.

**Figure 1:**
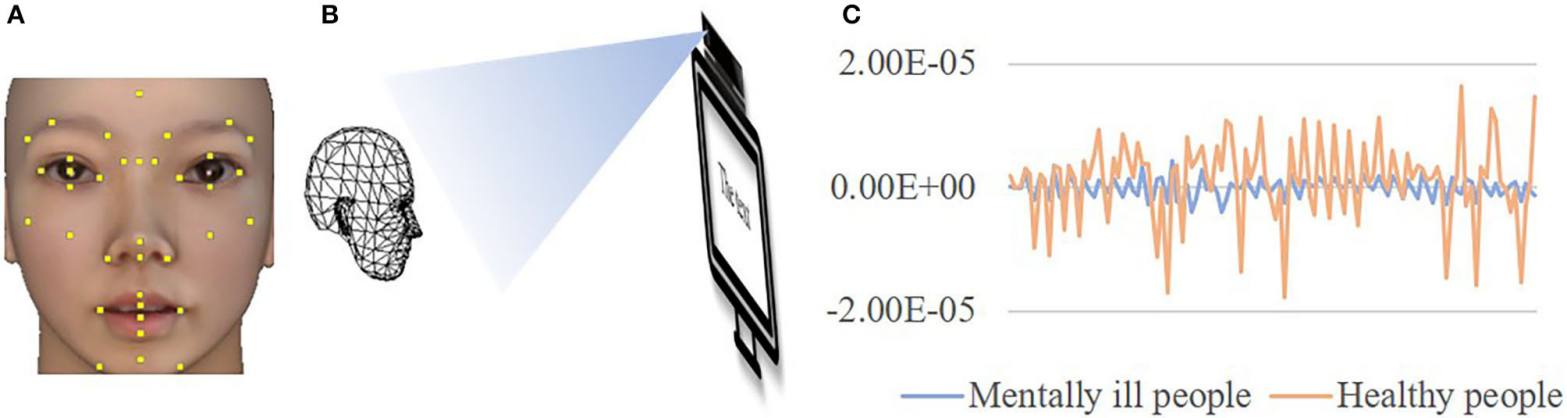
The participant's facial movements were recorded by Kinect. **(A)** The positions of the 36 facial key points on faces. **(B)** A Kinect camera recorded the facial movements of participants when they were reading a neutral text. **(C)** Time-series characteristics were extracted to discover the difference in facial movements between individuals with mental illnesses and those who were healthy. The face image was virtualized.

*Kinect*. Kinect is a low cost, convenient, and reliable depth sensor with an RGB image camera developed by Microsoft. Unlike traditional planar image characteristics, Kinect can record the movement of facial key points in 3D space (36). Therefore, comprehensive information about facial movements can be extracted. In this study, Kinect was purchased and Kinect for Windows SDK v2.0 was installed to record the 3D coordinates of key points on the face. Kinect can recognize 1,347 key points on the face, and key points near the facial features were considered to be the most closely related to mental illnesses such as depression (37). On this basis, we selected the points near the facial features and the center points of other parts as the key points for identifying mental health symptoms, which totaled 36. The positions on the face are shown in Figure 1A.

### Procedure

**Figure 2:**
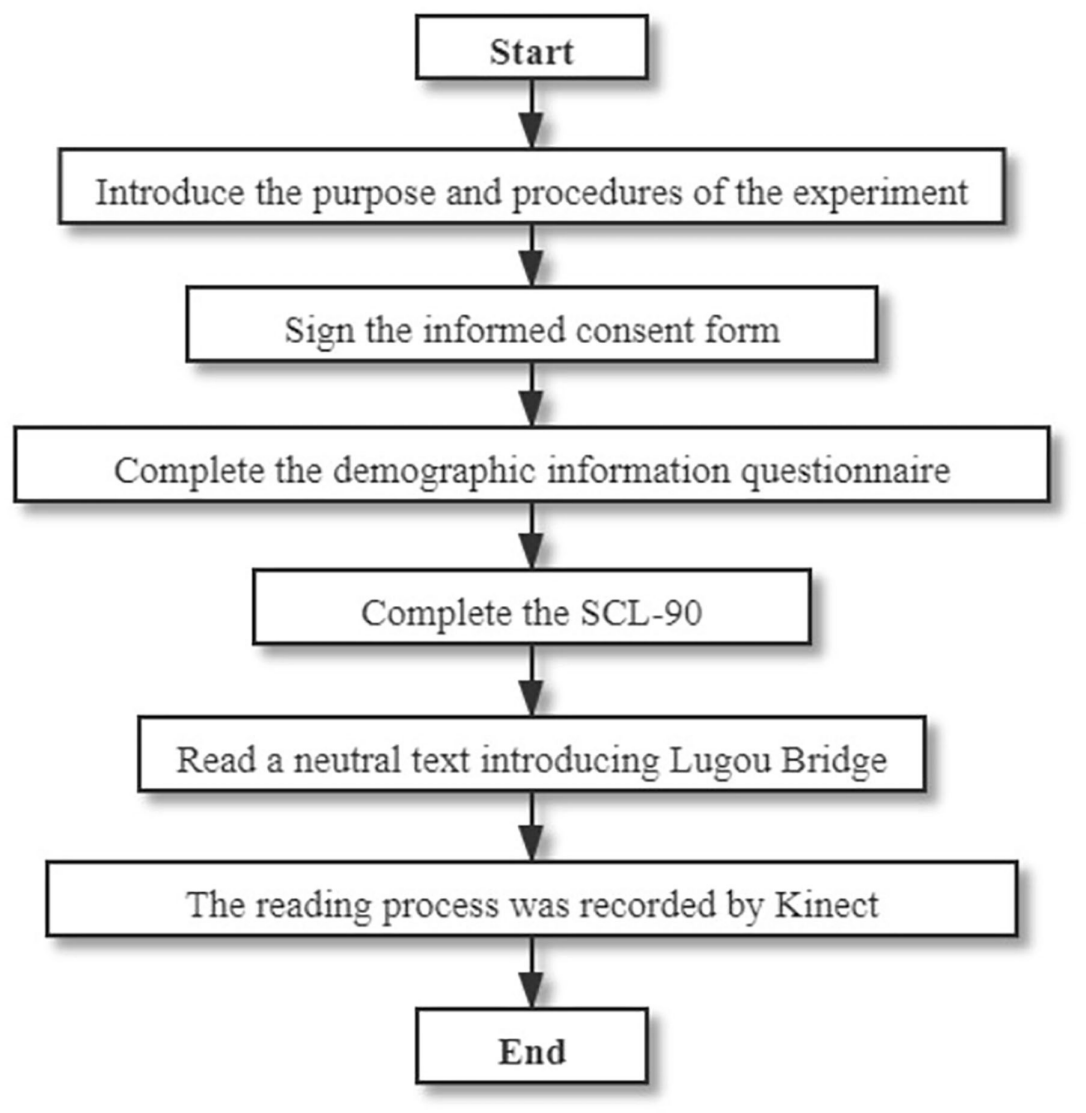
Data collection process.

*Data collection*. Participants were first asked to complete the demographic information questionnaire and the six subscales of the SCL-90. Then they read a neutral text introducing the Macro Polo bridge, during which Kinect was used to record their facial key point locations over approximately 30 s. The frame rate of Kinect is 30 HZ, the resolution of the captured image is 1,920 × 1,080 in color and 512 × 424 in depth (38). The distance between Kinect and the participant's seat was controlled to be 1.5 m to exclude the influence of distance on the intensity of facial movements. Meanwhile, we asked the participants to stay as still as possible in the instruction. The data collection for facial movements (as shown in Figure 1B), demographic information, and the SCL-90 were conducted according to the process shown in Figure 2.

**Figure 3:**
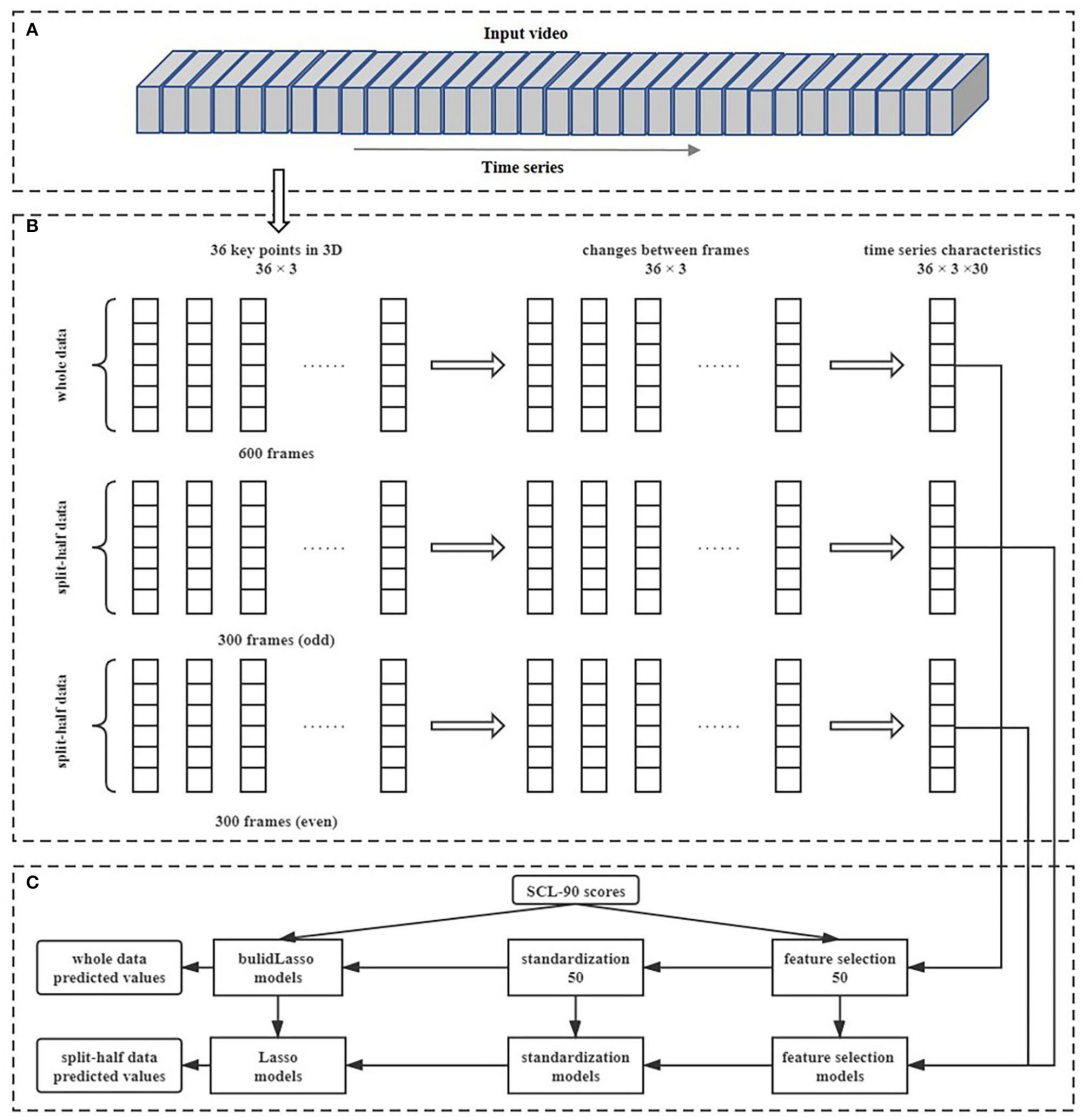
The process of data preprocessing, feature extraction, feature selection, and model building. **(A)** Represents the facial changes in the sequence over a period of time; **(B)** shows the process of data preprocessing and feature extraction; **(C)** shows the process of feature selection and model training.

*Data preprocessing*. After data collection, the scores of the subscales in the SCL-90 were calculated. For each participant's facial key point coordinate data, data preprocessing was conducted to eliminate the influence of noise. First, for each frame, we translated the origin of the key point coordinates to the position of key point 0 to balance the influence of the head movements. Then, for each frame, the average coordinates of the current frame, the previous frame, and the next frame were used as the coordinates of the current frame to balance the influence of noise. Next, we intercepted the data from the 100th frame to the 700th frame to eliminate the preparation time before and after reading (as seen in Figure 3A), which was approximately 20 s. Finally, we conducted a subtraction between the adjacent data in the time-series to obtain the coordinate changes. We named the 100th to 700th frames “whole” data, and the odd 300 frames and even 300 frames in the 600 frames “split-half” data.

*Feature extraction*. So that facial movements could be expressed as changes in the coordinates of key points, time-series characteristics were used to describe the movements of each key point in 3D space over time. The present study used 30 time-series characteristics as features to extract the motion information of facial key points across the entire time series. The names, types, and meanings of these 30 time-series characteristics are shown in Table 2. After feature extraction, we created a feature file, with each row for a participant and each column for a feature. Therefore, the feature file had 3D × 36 key points × 30 time-series characteristics = 3,240 columns. For example, a participant with mental illnesses had 108 (3 ^*^ 36) average values for the coordinate changes like the blue line in Figure 1C, while a healthy participant had 108 average values for the coordinate changes like the orange line in Figure 1C. As we can see in Figure 1C, some time-series characteristics can distinguish between individuals with mental illnesses and healthy individuals very well. Regardless of “whole” data or “split-half” data, the same features were extracted.

**Table 2:** The names, data types, and meanings of the time-series characteristics used in this study.

**Name**	**Type**	**Meaning**
Maximum	Float	The highest value of x
Minimum	Float	The lowest value of x
Mean	Float	The mean value of x
Variance	Float	The variance value of x
Std	Float	The standard deviation of x
Skewness	Float	The sample skewness of x
Kurtosis	Float	The kurtosis of x
Median	Float	The median of x
Absolute energy	Float	The sum over the squared values of x
Absolute sum of changes	Float	The sum over the absolute value of consecutive changes in x
Variance larger than std	Bool	If variance is greater than std
Count above mean	Float	The number of values in x that are higher than then mean of x
Count below mean	Float	The number of values in x that are lower than then mean of x
First location of maximum	Float	The first location of the maximum value of x
Last location of maximum	Float	The relative last location of the maximum value of x
First location of minimum	Float	The first location of the minimum value of x
Last location of minimum	Float	The relative last location of the minimum value of x
Duplicated	Bool	If any value in x occurs more than once
Max duplicated	Bool	If the maximum value of x is observed more than once
Min duplicated	Bool	If the minimum value of x is observed more than once
Longest strike above mean	Float	The longest consecutive subsequence in x that is bigger than the mean of x
Longest strike below mean	Float	The longest consecutive subsequence in x that is smaller than the mean of x
Mean absolute change	Float	The mean over the absolute differences between values in x
Mean change	Float	The mean over the differences between values in x
Percentage of reoccurring datapoints	Float	The percentage of unique values that are present in x more than once
Ratio value number	Float	The percentage of unique values that are present in x only once in all values
Sum of reoccurring datapoints	Float	The sum of all data points, that are present in x more than once
Sum of reoccurring datapoints	Float	The sum of all values, that are present in x more than once
Sum values	Float	The sum of all values
Range	Float	The range value of x

*x represents the time-series; std, standard deviation*.

**Figure 4:**
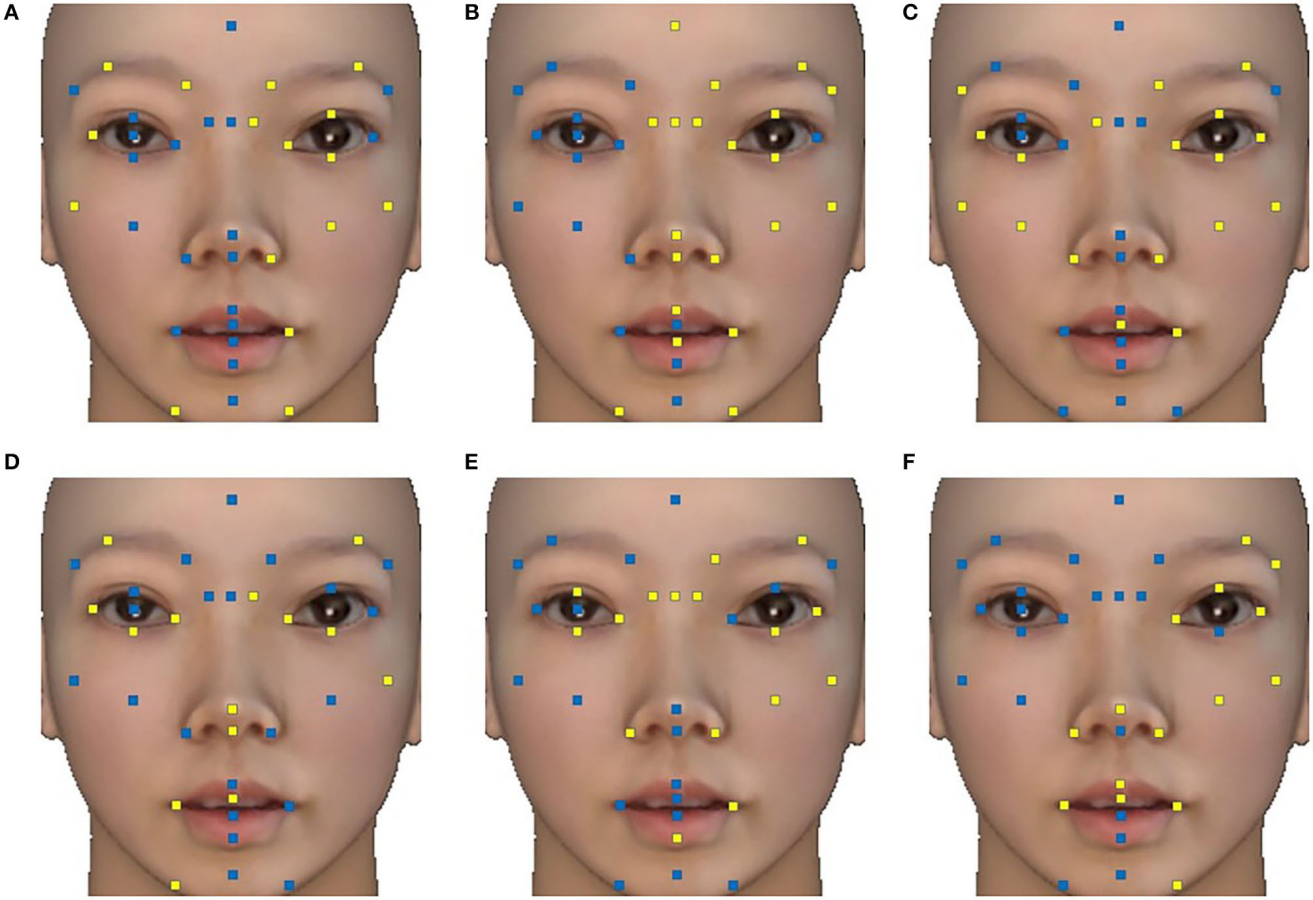
The key points that changed the most with the scale scores in each symptomatic dimension. Key points with at least one time-series characteristic included in the 50 features are marked in blue, and others are marked in yellow. **(A)** Interpersonal sensitivity; **(B)** depression; **(C)** anxiety; **(D)** hostility; **(E)** phobic anxiety; **(F)** psychoticism. The face images were virtualized.

*Feature selection*. After extracting 3,240 features for each participant, supervised feature extraction was used to select features that were “important” for each model, which were also features related to the subscale scores. *F*-values were calculated between each feature value for “whole” data and each dimension score. Finally, we selected the 50 features with the largest *F*-value for each model. The points that changed the most with the scores for each subscale are shown in Figure 4. It can be seen that the left side of the face expresses more information about mental health status than the right in most symptomatic dimensions of the SCL-90. The rules for selecting features were saved and used in the “split-half” data. After that, all features were standardized to ensure that the contribution of features to models was not affected by range and distribution.

*Model training*. Based on prior knowledge provided by other studies, the range of nonverbal activities is mostly linear with the degree of mental health (14), so the linear regression model was selected. Because too many features may lead to overfitting, we used L1 regularization to simplify the model. The least absolute shrinkage and selection operator (LASSO) (39) is an optimized technique in linear regression models which uses the L_1_-norm penalty. Equation 1 is a general representation of the objective function of LASSO regression, in which *y* represents the outcomes and *x* represents the features, *N* and *p* are the numbers of samples and variables, and λ and β are the adjustment parameters and regression coefficients. Compared with traditional linear regression models, LASSO regression can enhance the generalization ability of models (40). In this study, LASSO regressions were used to fit the linear relationship between features and subscale scores, and five-fold cross-validation was used to adjust model parameters. After cross-validation, all samples were predicted once as test sets, and the results were saved as predicted values. Similarly, we first used the “whole” data to build the models for each symptomatic dimension and then applied the models to the “split-half” data. The overall process is shown in Figure 3. Finally, we obtained three sets of predicted values with a number of 100 based on the “whole” data and “split-half” data.

(1) $$\begin{array}{r} \overset{N}{\underset{i=1}{\sum }}{\left(y_{i}-\underset{j}{\sum }x_{ij}\beta_{j}\right)}^{2}+\lambda \overset{p}{\underset{j=1}{\sum }}|\beta_{j}| \end{array}$$

### Statistical Analysis

For descriptive analyses of the quantitative variables, the mean and standard deviation values were calculated. Because of the large sample size and approximate normality distribution, a *t*-test was used to examine the differences in age and the SCL-90 scores between the mentally ill group and the healthy group. For analyses of the qualitative variables, the frequencies were used and chi-square tests were carried out to test differences in marital status, number of children, education level, and gender between the mentally ill group and healthy group. Predicted scores using “whole” data were defined as the predicted values for this method. The predicted scores of the “split-half” data were used as the “split-half” scores. The split-half reliability for each model was assessed with correlation coefficients between the “split-half” scores. Multitrait-multimethod matrix analysis and criterion validity analysis were conducted to test validity.

## Results

### Demographic Information

The demographic information of individuals was collected in this study. Participants in this study were middle-aged people with an average age of 40 years, they were mostly married (87%), and had children (82%). The proportion of participants who had received higher education was 57%.

### SCL-90 Score

The average value of the total scores of the SCL-90 was 88.13, and the standard deviation value was 24.03. Participants were divided into a “healthy group” (*n* = 88) and a “mentally ill group” (*n* = 12) based on the aforementioned threshold score of 110 points. Although the numbers of healthy subjects and mentally ill subjects are uneven, the data distributions of the total scores and the subscales scores are close to the normal distribution, which has less influence on the regression models. The demographic information was not distinguished between the two groups, except for gender. The scores of the six subscales were significantly different in the two groups, which was in line with expectations (see Table 3).

**Table 3:** Distribution of demographic information and SCL-90 scale scores.

	**Total**	**Healthy people (total score <110)**	**Mentally ill people (total score > = 110)**	***P-*value**
Sample size	100	88	12	
Age	40.23 (7.582)	40.51 (7.58)	38.17 (7.66)	0.731
Sex (male)	60	49	11	0.017^*^
Sex (female)	40	39	1	
Marriage (yes)	87	77	10	0.708
Children (yes)	82	72	10	0.932
Higher education (yes)	57	52	5	0.253
SCL-90	88.13 (24.03)	82.28 (13.91)	131 (37.24)	0.001^***^
Interpersonal sensitivity	16.03 (4.72)	15.08 (3.52)	23 (6.47)	0.000^***^
Depression	22.02 (6.558)	20.40 (4.01)	33.92 (9.199)	0.000^***^
Anxiety	15.19 (4.59)	14.25 (3.00)	22.08 (7.70).	0.000^***^
Hostility	9.96 (3.45)	9.24 (2.51)	15.25 (4.73)	0.001^***^
Phobic anxiety	9.24 (2.98)	8.65 (1.90)	13.58 (5.28)	0.008^**^
Psychoticism	15.69 (4.68)	14.67 (3.01)	23.17 (7.47)	0.002^**^

*Continuous data are expressed as mean (standard deviation); discrete data are expressed as number*.

* P < 0.05;

** P < 0.01;

*** *P < 0.001*.

### Split-Half Reliability

In this study, the original “whole” data was divided into two parts based on the parity of frames. And the Pearson correlation coefficient between the predicted values of the two split-half data was calculated as an indicator of split-half reliability. The split-half reliability of the six facial prediction models is shown in Table 4, all reaching the significance level.

**Table 4:** Split-half reliability and criterion validity of each dimension.

**Dimensions**	***R_**1**_***	***R_**2**_***
Interpersonal sensitivity	0.817^***^	0.377^***^
Depression	0.755^***^	0.261^**^
Anxiety	0.551^***^	0.307^**^
Hostility	0.516^***^	0.423^***^
Phobic anxiety	0.674^***^	0.351^***^
Psychoticism	0.608^***^	0.376^***^

*R_1_, the Pearson correlation coefficients between the predicted values of odd frames data and the predicted values of even frames data. R_2_, the Pearson correlation coefficients between the predicted values of the “whole” data and the actual score of the dimension scale*.

** P < 0.01;

*** *P < 0.001*.

### Convergent Validity and Discriminant Validity

This study used a multitrait-multimethod matrix to explore the structural validity of facial prediction models. Six traits were involved in the multitrait-multimethod matrix, which were interpersonal sensitivity, depression, anxiety, hostility, phobic anxiety, and psychoticism; and two methods were involved, including the SCL-90 subscales and facial prediction models. Pearson correlation coefficients were calculated among the predicted values and the SCL-90 scores, and Table 5 presents the zero-order correlation matrix between variables. In Table 5, the bold numbers on the diagonal represent the correlations between different methods measuring the same trait, the numbers in the triangles represent the correlations between different traits measured by the same method, and the numbers in the yellow area represent the correlations between different methods measuring different traits. The results indicated that the bold numbers were significantly larger than the data in the yellow area in the same column, except for the depression dimension, which meant that our models had good convergent validity. However, the bold numbers were not all greater than the corresponding values in the triangles, which meant the discriminant validity of our models was not as good.

**Table 5:** Convergent validity and discriminant validity of each dimension.

		**Facial prediction models**						**SCl-90**					
		**INT_**1**_**	**DEP_**1**_**	**ANX_**1**_**	**HOS_**1**_**	**PHO_**1**_**	**PSY_**1**_**	**INT_**2**_**	**DEP_**2**_**	**ANX_**2**_**	**HOS_**2**_**	**PHO_**2**_**	**PSY_**2**_**
Facial prediction models	INT_1_												
	DEP_1_	0.74											
	ANX_1_	0.42	0.44										
	HOS_1_	0.39	0.27	0.29									
	PHO_1_	0.48	0.49	0.43	0.23								
	PSY_1_	0.49	0.48	0.50	0.16	0.27							
SCL-90	INT_2_	**0.38**	0.29	0.25	0.20	0.19	0.34						
	DEP_2_	0.35	**0.26**	0.22	0.29	0.18	0.32	0.73					
	ANX_2_	0.27	0.21	**0.31**	0.23	0.22	0.27	0.75	0.81				
	HOS_2_	0.23	0.14	0.16	**0.42**	0.08	0.21	0.64	0.77	0.71			
	PHO_2_	0.26	0.18	0.24	0.18	**0.35**	0.16	0.64	0.74	0.74	0.63		
	PSY_2_	0.33	0.28	0.25	0.23	0.26	**0.38**	0.82	0.82	0.84	0.68	0.72	

*The Pearson correlation coefficients between the predicted values of each model and the actual scores of each scale. INT, interpersonal sensitivity; DEP, depression; ANX, anxiety; HOS, hostility; PHO, phobic anxiety; PSY, psychoticism; 1, predicted values by facial prediction models; 2, actual scores by the SCL-90*.

### Criterion Validity

The actual scores of each subscale were used as the effective standard, and the Pearson correlation coefficients between the predicted values of the “whole” data and the actual scores of the corresponding subscales were calculated, so as to conduct the analysis of criterion validity (as shown in Table 4). The results showed that the correlation coefficients had reached a significant level, which meant the models established had high criterion validity.

## Discussion

The present study tested the prediction of psychological symptoms based on facial movements. We collected SCL-90 scale scores as the output, and extracted the time-series characteristics of facial key points as the input, then built facial prediction models for each symptomatic dimension. Finally, we tested the stability and availability of the models by calculating the split-half reliability, criterion validity, convergent validity, and discriminant validity. The results indicated that the facial prediction models proposed have good split-half reliability, criterion validity, and convergent validity, although the discriminant validity is lower.

Consistent with previous research on emotion-induced situations (41, 42), the high criterion validity suggests that under neutral conditions, facial movements can also be used to distinguish patients with mental illness from those who are healthy, especially the facial movements on the left side of the face. This finding is in line with previous studies that found that individuals with some mental illnesses have fewer facial movements than healthy people due to alexithymia (43, 44). An alternative explanation would be that compared with healthy people, people with poorer mental health status are more likely to produce (45) and express (46) negative emotions under neutral stimulation. Although each model had significant criterion validity, it is noteworthy that the depression model and anxiety model had lower criterion validity than the other symptomatic dimensions. Based on previous studies, we speculate that this is because comorbidity with anxiety or depression is common in people with other symptoms (47, 48). Individuals with depression and anxiety may have different subtypes, which leads to different facial movements and results in slightly lower criterion validity. Relevant studies have also pointed out that there are differences in the performance of individuals with multiple symptoms and those with only depression or anxiety (49, 50). One possible explanation for the finding that the left side of the face is more capable of expressing mental health status is that mental illness, such as depression and autism, are mainly dominated by the right hemisphere of the brain (51).

There was also fairly high convergent validity for most models except depression. Specifically, for the interpersonal sensitivity dimension, anxiety dimension, hostility dimension, phobic anxiety dimension, and psychoticism dimension, the correlations between different methods measuring the same traits were higher than all the correlations between different methods measuring different traits, which meant the two methods were measuring the same traits, consistent with our expectations. However, in the depression dimension, we did not find a higher correlation between different methods measuring the same trait, which indicates that the depression dimension may not have a specific facial expression that can be identified, and this is probably related to the complex comorbidity between depression and other negative psychological symptoms (47, 52, 53). Studies have suggested that different types of negative mental health status have different facial movements (54, 55) and the facial expressions associated with mental illness are also different from physical illness (56, 57). Our study suggests the possibility that different psychological symptoms of mental illnesses may have different facial movements that can correspond to the SCL-90 scores, which are detailed and granular. Future study is needed to explore the unique expression of each symptomatic dimension and the underlying neurological mechanisms. In addition, it is understandable that the discriminant validity is low, considering the high correlation (0.3–0.8) between the scores of the various subscales in the SCL-90 (58), and the high correlation (0.2–0.7) between the values of models which are based on scale scores.

In terms of reliability, results indicate good levels of split-half reliability for all the models (from 0.52 to 0.82), which are consistent with the subscale consistency (from 0.50 to 0.90) (59–61) in previous studies examining the SCL-90. The credible split-half reliability suggests that the time-series characteristics we extracted can represent stable personal traits to some extent, rather than random factors. One previous study has explored the stability within individuals and differences between individuals in facial expressions (62). Such differences may relate to mental health status and other individual characteristics, and such stability may be the reason why the machine learning models have good reliability.

Our study indicates that the facial prediction models based on the SCL-90 have good split-half reliability, criterion validity, and convergent validity. As per the literature explored and to the knowledge of the authors, we are the first to measure the reliability and validity of machine learning models. In multi-dimensional studies, measuring the reliability and validity of machine learning models is conducive to ensuring one model can truly discover the pattern of the corresponding symptomatic dimension, which cannot be achieved by previous machine learning evaluation methods.

Our research also provides a feasible method for evaluating the performance of multi-trait machine learning models. The multi-dimensional psychological symptoms of mental health were predicted separately in this study, and most models had satisfactory convergent validity, which presents the possibility of predicting more detailed aspects of mental health through the assessment of facial movements. Furthermore, we tracked the facial movements of participants under neutral stimulation, which is close to the facial state of people during normal communication. Although the current facial prediction models cannot replace scales, existing research could be combined with monitoring technology to achieve large-scale and non-invasive mental health monitoring for appropriate occupations in practical applications.

This study also has some limitations. First, the selection of the machine learning algorithm should ensure that it can match the corresponding dataset. Selecting deep learning algorithms may slightly improve the results, but this is not the focus of this paper. Future studies based on different datasets would be needed to compare the performance of different machine learning models. In addition to regression models, classification prediction models are also of practical significance, as long as the data are balanced. Second, considering the purpose of the research, we used the SCL-90, of which the correlation among the subscales was very high. This results in low discriminant validity. Further work should take into account the comorbidity between symptoms and strive to obtain a unique facial expression for each symptom. Third, as the participants in this study were conveniently sampled at a large-scale event, although age and gender were balanced, the specific occupation of the participants may also cause some sampling bias. Moreover, due to limited time, the three symptoms of somatization, compulsion, and paranoia were not measured, and those symptoms could be explored in further studies. A further limitation may be the influence of participants' knowledge background in self-reporting methods. However, in our data acquisition and application scenarios, self-reporting was the most appropriate method. Future research can try to use the diagnosis of psychiatrists as the annotation data of prediction models. Finally, the criterion validity of the depression and anxiety models was lower compared with other models. Future research can try different data collection scenarios and feature extraction methods to better predict the psychological symptoms with many subtypes.

## Conclusion

We proposed facial prediction models based on the SCL-90 and demonstrated that the measurement has high reliability and satisfactory validity. Furthermore, this study demonstrated that facial movements can distinguish multi-dimensional psychological symptoms, and provides a feasible method to evaluate the performance of multi-trait machine learning models.

## Data Availability Statement

The datasets generated for this article are not readily available because the raw data cannot be made public, if necessary, feature data can be provided. Requests to access the datasets should be directed to liuxiaoqian@psych.ac.cn.

## Ethics Statement

The studies involving human participants were reviewed and approved by the scientific research ethics committee of the Chinese Academy of Sciences Institute of Psychology (H15010). The patients/participants provided their written informed consent to participate in this study. Written informed consent was obtained from the individual(s) for the publication of any potentially identifiable images or data included in this article.

## Author Contributions

TZ contributed to the conception and design of the study. XL collected the data and developed the instrument. BL provided guidance for data preprocessing and model establishment. MZ provided guidance for the reliability and validity testing plan. YW performed the statistical analysis. XW trained the facial prediction models and wrote the manuscript with input from all authors. All authors contributed to the article and approved the submitted version.

## Conflict of Interest

The authors declare that the research was conducted in the absence of any commercial or financial relationships that could be construed as a potential conflict of interest.
